# Effector–Immunity Pairs Provide the T6SS Nanomachine its Offensive and Defensive Capabilities

**DOI:** 10.3390/molecules23051009

**Published:** 2018-04-26

**Authors:** Xiaobing Yang, Mingxiu Long, Xihui Shen

**Affiliations:** 1 Shaanxi Key Laboratory of Agricultural and Environmental Microbiology, College of Life Sciences, Northwest A&F University, Yangling 712100, China; bing3698@163.com; 2 College of Animal Science and Technology, Northwest A&F University, Yangling 712100, China; longmingxiu@nwsuaf.edu.cn

**Keywords:** types VI secretion systems (T6SS), effector–immunity pairs (E–I pairs), protein-protein interaction (PPI), interbacterial competition

## Abstract

Type VI protein secretion systems (T6SSs) are specialized transport apparatus which can target both eukaryotic and prokaryotic cells and play key roles in host–pathogen–microbiota interactions. Therefore, T6SSs have attracted much attention as a research topic during the past ten years. In this review, we particularly summarized the T6SS antibacterial function, which involves an interesting offensive and defensive mechanism of the effector–immunity (E–I) pairs. The three main categories of effectors that target the cell wall, membranes, and nucleic acids during bacterial interaction, along with their corresponding immunity proteins are presented. We also discuss structural analyses of several effectors and E–I pairs, which explain the offensive and defensive mechanisms underpinning T6SS function during bacterial competition for niche-space, as well as the bioinformatics, proteomics, and protein–protein interaction (PPI) methods used to identify and characterize T6SS mediated E–I pairs. Additionally, we described PPI methods for verifying E–I pairs.

## 1. Introduction

Bacteria interact with the environment and with other bacteria by means of secreted proteins, and have evolved multiple protein transportation pathways including the secretion systems [1,2,3,4]. To date, seven types of bacterial protein secretion systems (Types I–VII, or T1SS–T7SS) have been described [3]. These secretion systems deliver effector proteins either into the extracellular milieu or into eukaryotic or prokaryotic cells, and play crucial roles in bacteria adaptation to their environments, thwarting host defense, and acquisition of important nutrients [5,6]. The bacterial Type VI Secretion System (T6SS) is a widely distributed contact-dependent apparatus with structural and morphological similarities to contractile phage tails [7,8]. Some T6SSs associated with pathogens are necessary for full virulence by injecting anti-eukaryotic effectors into host cells to modulate host immunity [9], facilitate eukaryotic membrane fusion [10], and interact with the microtubule network [11]. However, the best-characterized function of T6SSs is delivery of antibacterial toxins, such as muramidases or glycoside hydrolases that attack the bacterial cell wall [12,13,14], nucleases that target nucleic acids [15], and lipases that degrade membrane structures [16], into prokaryotic cells to facilitate inter-species competition. These toxic effectors play offensive roles not only for adjacent cells but also for bacteria themselves. In order to protect themselves and sister cells from secreted effector intoxication, bacterial killing T6SS^+^ organisms will often express cognate immunity proteins to neutralize the toxicity of antibacterial effectors [12,17,18]. As the effector–immunity (E–I) pairs seem crucial during bacterial competition, in this review, we will summarize the known E–I pairs of T6SS.

## 2. Effector–Immunity (E–I) Pairs of T6SS

Bioinformatics approaches have successfully identified one or more T6SS clusters in approximately 25% of Gram-negative bacteria, including many important pathogens [5,19]. Currently, the T6SS apparatus is considered a bacterial nanoweapon, composed of approximately 13 conserved core components (TssA–M) that form a single cell-puncturing tool [20,21,22,23]. Usually, the tail tube (haemolysin coregulated protein, Hcp/TssD) and spike complex (valine-glycine repeat protein G, VgrG/TssI) which are injected into neighboring cells or the extracellular milieu, are considered the hallmark of functional T6SS apparatus [7,24,25,26,27]. While initially T6SSs were reported to target eukaryotic cells [24,28], accumulating studies demonstrate that T6SSs were primarily used as a weapon to kill competing bacterial species [17,29,30,31]. The antibacterial functions of T6SSs are attributable to secreted antibacterial effector toxins, which are neutralized in secretor strains by corresponding antagonistic immunity proteins that prevent self-killing or sibling-intoxication. Thus, E–I pairs constitute a new toxin-antitoxin module, providing sister cells with protection against toxic effectors [12,17,32,33].

The first work identifying E–I pairs was reported by Hood *et al* in 2010. Three type six exported effectors (Tse1–3) were found to be secreted by H1-T6SS in *Pseudomonas aeruginosa*, and Tsi2 is a cytoplasmic immunity protein that physically interacts with the Tse2 to protect *P. aeruginosa* from Tse2 toxicity [17]. Following this initial work, a number of T6SS E–I pairs have been identified in a variety of bacterial species, and it has been found that antibacterial effectors can be divided into three categories according to their targets: the cell wall, cellular membranes, and nucleic acids [34,35,36]. Recently identified E–I pairs are listed in Table 1.

### 2.1. Cell Wall Targeting Effectors

In 2011, it was shown that Tse1 and Tse3, secreted by the H1-T6SS apparatus in *P. aeruginosa*, play crucial roles in interspecies competition by degrading peptidoglycan via amidase and muramidase activity. Secretor cells are protected by immunity proteins (Tsi1 and Tsi3), which specifically interact with and inactivate the cognate toxins in the cellular periplasm [32]. In *Vibrio cholerae*, the C-terminal extension of VgrG-3 (VgrG-3C) was found to degrade peptidoglycan and hydrolyse the cell wall of Gram-negative bacteria, and the TsaB (type six secretion antitoxin B) was identified as the immunity protein [18,37].

Currently, cell wall-degrading T6SS effectors are divided into two groups; the first comprising amidases or muramidases, which cleave the peptidoglycan molecule within its peptide stems and cross-links, and the second containing glycoside hydrolases, which cleave the glycan backbone of the molecule [13]. Based on sequence homology and substrate specificities, 51 amidase E–I pairs, in four highly divergent families (Tae1–4), have been described. The aforementioned Tse1/Tsi1 is also defined as Tae1/Tai1, belonging to family 1 [14,38]. Novel families of T6SS peptidoglycan glycoside hydrolase effectors (Tge proteins) have recently been discovered. For example, a Tge from *Pseudomonas protegens* displays periplasmic toxicity, and confers a fitness advantage against another co-occurring soil bacterium, *Pseudomonas putida*, while preventing self-intoxication with the immunity protein Tgi through direct occlusion of the active site on Tge [13].

Several crystal structure studies have been conducted in order to dissect the offensive and defensive mechanisms of T6SS E–I pairs. Analysis of the crystal structure of Tsi1 from *P. aeruginosa* revealed a unique structural feature of five loops (The HI, CD, EF, JK, and LM loops) by which Tse1 could be distinguished from other NlpC/P60 family members, and which allowed binding sites to be matched. The interaction between the five loops of Tsi1 and the 14 residues of Tse1 forms a hydrogen-bond network, the formation of which blocks the binding of Tse1 to peptidoglycan [39,40,41]. The crystal structure of the Tae3-Tai3 complex in *Ralstonia pickettii* revealed a stable Tae3-Tai3_4_-Tae3 heterohexamer, in which inhibition of effector activity is achieved through the physical insertion of the Ω-loop of Tai3 into the active-site cleft of Tae3 [42]. It is suggested that, in *P. aeruginosa*, the Tse3 and Tse3-Tsi3 complex anchors to the outer membrane in a calcium-dependent manner, with a Y-shaped groove on the surface of the Tse3 C-terminal domain serving as the peptidoglycan binding site. Tsi3 is likely inserted into the substrate-binding groove of Tse3 to form the Tse3-Tsi3 complex, thereby inhibiting Tse3 activity [43]. Structure-based mutational and crystal structure analysis of Tae4-Tai4 complexes from the human pathogens *Salmonella typhimurium* and *Enterobacter cloacae* revealed that a helix (α3) of one subunit in dimeric Tai4 is crucial for Tae4-Tai4 binding, and that a protruding loop (L4) in the other subunit is mainly responsible for Tae4 activity inhibition [33]. Further studies revealed cross-neutralization between *St*Tae4 and *Ec*Tai4, suggesting that the inhibition mechanism is conserved within the two species [44]. The antibacterial amidase Tae4 secreted by T6SS of the enteropathogenic bacterium *Salmonella enterica* serovar Typhimurium could kill *Klebsiella oxytoca* in the host gut in an Hcp1-dependent manner. This finding indicates that the T6SS antibacterial weapon is necessary for bacteria to establish infection within the host gut [45]. 

Representing another family, Ssp proteins were identified as special antibacterial effectors which, together with their cognate immunity Rap (resistance-associated protein) proteins, are encoded within the T6SS gene cluster in *Serratia marcescens*. Biochemical analyses demonstrated that these effectors belong to new protein families, and that the structure of the Rap protein is dependent on the formation of a disulphide bond which had not been previously described [46]. In a follow-up study, Ssp1 and Ssp2 peptidoglycan endopeptidase activity was characterized, with structural analysis revealing the neutralization specificity of Ssp1-Rap1a and Ssp2-Rap2a complexes [47]. In addition to the predicted amidases Ssp1 and 2, further proteins (Ssp3–6) found in *S. marcescens* Db10 were identified as antibacterial toxins with unknown function [48].

### 2.2. Membrane Targeting Effectors

VasX is present in *V. cholerae* as a novel membrane-localized T6SS virulence factor that interacts with membrane lipids [49]. Using a transposon insertion-site sequencing (Tn-seq) method, three immunity proteins, which in *V. cholerae* render protection against killing by T6SS predatory cells, together with corresponding T6SS effectors (VasX, TseL, and VgrG3), were identified. TseL was found to possess a lipase domain, suggesting that its activity is also membrane-targeted. Inactivation of a conserved residue located in its active site abolished its toxic activity as a T6SS effector. VgrG3 possesses a C-terminus peptidoglycan-binding domain (PBD), and is capable of disrupting the integrity of the bacterial cell wall [12]. These three effectors and their associated immunity proteins were confirmed in a later study, in which it was demonstrated that all three immunity proteins are controlled in a dual fashion, wherein both are regulated by the promoters upstream of the T6SS clusters and the individual regions with promoter activity that drive expression of immunity protein-encoding genes within T6SS clusters. This dual expression profile of T6SS immunity genes provides pandemic *V. cholera* strains with T6SS immunity, preventing T6SS-silent strains from killing neighboring bacterial competitors [50].

Based on an analysis of 377 putative T6SS-mediated lipases, type VI secretion lipase effectors (Tle) are divided into five divergent families (Tle1–5) [16]. In *Escherichia coli* EAEC 17-2, Tle1 possesses phospholipase A_1_ and A_2_ activities and is responsible for antibacterial activity during interbacterial competition. Self-protection of the attacker cells is affected by Tli1, an outer membrane lipoprotein which inhibits phospholipase activity by directly binding to Tle1 [51]. In *P. aeruginosa*, PldB (Tle5b) was characterized as a trans-kingdom H3-T6SS dependent phospholipase D effector that influences prokaryotic cells and eukaryotic hosts, respectively, targeting the periplasm or activating the PI3K/Akt pathway. Three cognate immunity protein genes are encoded downstream of *pldB*, ORFs PA5088, PA5087, and PA5086 [52]. Similarly, the H2-T6SS mediated trans-kingdom effectors PldA(Tle5) and TplE(Tle4) in *P. aeruginosa* both possess phospholipase activities, and their cognate immunity proteins are Tli5 and Tli4 [16,53].

PldA and PldB are the first examples of trans-kingdom effectors [52]. Both PldA and PldB target the host PI3K (phosphoinositide3-kinase)/Akt pathway, facilitating entry of *P. aeruginosa* into non-phagocytic epithelial cells [52,54]. TplE is the third trans-kingdom effector identified in *Pseudomonas aeruginosa*. TplE lipolytic activity contributes to intra- and interspecies competition, neutralized by the immunity protein Tli4. The TplE effector also contains a eukaryotic PGAP1-like domain, which targets and disrupts the host endoplasmic reticulum, leading to the activation of the unfolded protein response (UPR) through the IRE1α-XBP1 pathway, enhancing autophagic flux [53]. Similarly, both the TseL lipase and VasX in *V. cholerae* were also regarded as trans-kingdom effectors for their dual function in *Dictyostelium discoideum* amoeba killing and intraspecies bacterial competition [12,16,50]. This kind of E–I pair supports the pathogens to efficiently infect host cells without self-intoxication. The findings of trans-kingdom effectors provide new insights in understanding the mechanism of T6SS in pathogenesis, and other trans-kingdom effectors will be identified in the near future.

### 2.3. Effectors Targeting Nucleic Acids

Rhs (Rearrangement hot spot) proteins are large filamentous toxins with N-terminal regions exhibiting RHS repeats, whereas C-terminal regions are highly variable toxin domains [34,55]. The Rhs of *Dickeya dadantii* 3937 showed cell growth inhibitory activity when expressed in *E. coli*, demonstrating the role of Rhs in intercellular competition. Its toxic activity is specifically neutralized by cognate RhsI immunity protein [56]. Further study revealed that the role of Rhs proteins in intercellular competition is brought about by nuclease activity, which degrades target cell DNA in a contact-dependent manner. Immunity proteins (RhsI) could specifically neutralize cognate toxins to protect *rhs*^+^ cells from autoinhibition. It is noteworthy that *D. dadantii* 3937 *rhs* genes do not encode secretion signal sequences, and that secretion of Rhs proteins is linked to the VgrG component of T6SS [15]. The antibacterial T6SS of the opportunistic enteric pathogen *S. marcescens* Db10 plays crucial roles in intraspecies and interspecies competition [31]. Its T6SS-dependent antibacterial function relies on two Rhs proteins, one of which acts as DNase toxin, while the other contains a novel cytoplasmic-acting toxin domain. In this organism, Rhs proteins were reported to be the primary determinant in intraspecies competition [34]. Rhs-CT modules were found to be widely distributed as T6SS E–I pairs in *E. coli*, with diverse DNase, RNase, deaminase, and metallopeptidase activities. The antibacterial activities of Rhs-CT1, -CT3, and -CT5 were experimentally determined, and it was found that Rhs-CT1 is needed for intestinal colonization. It was further suggested that highly diverse Rhs-CTs could clinically and environmentally modulate microbial communities [20]. Recently, a toxic Rhs-type effector Tke2 secreted by the K1-T6SS in *Pseudomonas putida* was also identified, and the Tke2 toxicity is antagonized by the Tki2 immunity protein [57].

*Agrobacterium tumefaciens* uses the T6SS to repress or kill competitors during in plant colonization, and this antibacterial activity is mainly relying on Tde toxins. With a conserved HxxD motif, Tde shows antibacterial DNase activity distinct from previously known polymorphic toxins and nucleases. Tde toxicity is counteracted by a cognate immunity protein, Tdi. Based on BLASTP analysis, *tde–tdi* couples appear to be conserved in some Gram-negative proteobacteria, and are highly prevalent in a wide range of plant pathogens and plant growth-promoting bacteria [58].

Bioinformatics analyses revealed that Hcp proteins with diverse C-terminal extension toxins (defined as Hcp-ET) are widespread among the *Enterobacteriaceae*, and five Hcp-ETs, together with their immunity proteins, were characterized. The antibacterial Hcp-ET1 degrades target cell DNA via predicted HNH-DNase activity through T6SS2-dependent delivery. Hcp-ET2 possesses Tle1 phospholipase activity, and Hcp-ET3-4 is fused with Pyocin S3 and Colicin-DNase. Additionally, these Hcp-ETs toxin domains (HNH-DNase, DUF2235, Pyocin S3, and Colicin-DNase) are also widely present in diverse bacterial species [59].

### 2.4. Other Effectors and Their Chaperones

In *P. aeruginosa*, Tse2 induces quiescence within recipient cells, but the previously identified Tsi2 was not necessary for targeting Tse2 to the secretory apparatus. Crystal structure analysis showed that Tsi2 assembles as a dimer, which is not responsible for Tse2 immunity or antitoxin. Instead, an acidic patch distal to the Tsi2 homodimer interface was revealed to mediate the toxin interaction and immunity of Tse2 [60]. Determination of the Tse2/Tsi2 complex’s X-ray crystal structure revealed a heterotetrameric structure with an extensive binding interface. Tse2 showed NAD-dependent ADP-ribosyltransferase activity, and the Tse2 active site was occluded upon binding the inhibitor Tsi2 [61]. Tse4 has been identified in *P. aeruginosa* as an H1-T6SS-delivered antibacterial toxin by means of a quantitative cellular proteomics screen for T6SS substrates [62]. A recent report by LaCourse et al. showed that Tse4 is most active in high-salinity environments and synergizes with effectors that degrade the cell wall or inactivate intracellular electron carriers [63]. Another T6SS effector recently found in *P. aeruginosa*, Tse6, requires interaction with translation elongation factor Tu (EF-Tu) for delivery into target cells, where it acts by degrading β-nicotinamide adenine dinucleotide (NAD^+^) and NAD^+^ phosphate (NADP^+^) [64]. In a background deletion study of the Tse1–3 toxins in *P. aeruginosa*, RhsP-CT showed antibacterial toxicity against *E. coli*, which were protected by a cognate immunity RhsI [65]. In the human colon, *Bacteroides fragilis* 638R utilize two Bfe–Bfi pairs of GA3 T6SSs to antagonize most human gut *Bacteroidales* strains [66]. In *Aeromonas hydrophila*, the antibacterial toxicity of TseC/TsiC is attributed to the colicin domain on TseC [67].

Recently, a T6SS4-mediated effector in *Yersinia pseudotuberculosis*, YezP, was identified as a novel Zn^2+^-binding protein which provides resistance to oxidative stress and is partly responsible for bacterial pathogenicity [68]. Similarly, TseZ and TseM in *Burkholdeira thailandensis*, which are transported by T6SS4, exhibit Zn^2+^ and Mn^2+^ acquisition ability, respectively [69,70]. An H3-T6SS secreted effector, TseF, is incorporated into *P. aeruginosa* outer membrane vesicles (OMVs) where it contributes to iron acquisition by interacting with the iron-binding quinolone signal (PQS) system [71]. These studies revealed a new category of T6SS effectors whose ion-transporting functions enhance T6SS^+^ organisms’ ability to compete effectively in nutrient-limited environments.

## 3. Methods for Identifying T6SS E–I Pairs

As E–I pairs mediated by T6SS play several crucial roles in cell–cell interactions and provide self-protection for Gram-negative bacteria containing T6SS, multiple strategies and techniques have been employed in the identification of these pairs. A common strategy involves analysis of corresponding genes residing within or in close proximity to T6SS-encoding gene clusters [13,59,73]. Since the coding genes for E–I pairs are always adjacent on the bacterial genome [59], the effector and cognate immunity protein are often identified simultaneously. Methods used in the search for T6SS E–I pairs include: bioinformatic analysis, genetic analysis of T6SS-associated genes, mutant library screening, proteomics-based methods, and protein–protein interaction (PPI) methods [5,73].

**Bioinformaticsanalysis:** Informatics approaches are the primary method for examining the genetic context, sequence, and phylogenetic distribution of T6SS^+^ bacterial species. Several diverse superfamilies of bacterial T6SS effectors have been discovered using this approach. For example, on the basis of sequence homology and differing substrate specificities, the peptidoglycan peptidase effectors were classed into four highly divergent families (Tae1–4) [14]. Based on informatics forecasts and laboratory research, several web-based resources for identifying T6SS in bacteria have been established, including T346Hunter, SecReT6, and others, which may aid in the identification of putative E–I pairs around T6SS core components [74,75].

**Geneticanalysis of T6SS-associatedgenes:** Genetic analysis of T6SSs gene clusters contribute to the identification of new E–I pairs. For example, a VgrG3 C-terminal domain from *V. cholerae* (VgrG-3C) was identified as a peptidoglycan-targeting glycoside hydrolase [18,37]. Recently, a systematic search for extended Hcps in 17 bacterial species from the family *Enterobacteriaceae* revealed more than 350 Hcps C-terminal extension toxins (Hcp-ET domains), which could be classified into 5 clans: HNH-DNase, DUF2235, Pyocin S3, Colicin-DNase, and Papain-like peptidase [59]. In addition, by matching the diagnostic domain-architecture of an Rhs protein possessing an N-terminal PAAR motif and C-terminal toxin domain (Rhs-CT modules), several novel T6SS E–I pairs were identified [20].

**Mutantlibraryscreening:** Mutant library screening is a powerful phenotype-dependent tool for identifying T6SS effectors or cognate immunity proteins [73]. Because transposon insertion mutants of *vas* genes lose their anti-amoebae activity and display decreased Hcp secretion [28], the phenotype screening strategy seemed promising for identifying associated T6SS effectors [76]. As an immunity protein is required for resistance to wild-type sibling bacteria, three immunity genes in *V. cholerae* were identified by means of a transposon insertion-site sequencing (Tn-seq) method. The cognate T6SS effectors (TseL, VgrG3, VasX) were identified, confirming this approach [12]. However, this approach may be limited to the identification of non-cytoplasmic effectors.

**Proteomics-basedmethods:** Proteomics-based methods are a discovery-driven approach for identifying potential T6SS effectors when prior knowledge is absent. By means of comparative proteomics analysis of wild-type strains and strains with mutant T6SS apparatus, new enzymatic effectors have been identified in several bacteria [72]. For example, *P. aeruginosa* Tse1–3 effectors were identified by secretome analysis of mutants in which regulation of H1-T6SS had been altered. While the identification of immunity protein Tsi2 was through a self-intoxication assay, for the immunity protein does not need to be secreted out [17]. Slp, a VgrG-dependent effector in *S. marcescens*, was identified by performing similar secretome analyses on each specific *vgrG* mutant [77]. Proteomics analysis may be used in combination with other methods. For example, a new T6SS E–I pair, TseH–TsiH in *V. cholerae*, was identified using comparative proteomics coupled with bioinformatics [72].

**PPI methods:** The PPI methods are based on the interaction of substrates with T6SS injection components Hcp or VgrG. The tail tube and spike complex assembled by Hcp and VgrG is injected into neighboring cells or the extracellular milieu, and thus the associated effectors may be identified through correlation analysis of Hcp and VgrG. Hcp is depicted not only as a static conduit, but also an exported chaperone for T6SS substrates. The secretion of Tse2 in *P. aeruginosa* requires direct interaction with Hcp1 [78]. Similarly, T6SS toxins may also fuse to the VgrG spike, and subsequently be delivered upon sheath contraction [51]. On the tip of the VgrG spike, a special PAAR (proline-alanine-alanine-arginine) complex forms a sharp conical extension, which sharpens the tip and contributes to effector domain attachment to the VgrG spike [79,80]. Several PPI methods may also be exploited for substrate recognition through protein translocation techniques. These include yeast/bacteria two-hybrid screening, pull-down assays with glutathione-S-transferase (GST) or other tags, and co-immunoprecipitation (Co-IP). In the case of two-hybrid assays, if the expression of the effector toxin is harmful to the host, a few key effectors may be missed during screening. Furthermore, PPI methods can only recognize effectors that directly or tightly bind with adaptors/chaperones or with the T6SS machine [73].

## 4. Methods for Verifying the T6SS E–I Pairs

First of all, the antibacterial effector should be confirmed to be delivered via a specific T6SS machine and to mediate interbacterial killing, which means the following work would focus on a bonafide T6SS substrate. The next strategy for verifying E–I pairs involves testing whether candidate immunity proteins are capable of neutralizing the toxicity of the effectors in question. Co-expression and self-intoxication assays are commonly used methods. For co-expression methods, the effector gene is cloned into a specific vector, with or without the candidate immunity gene, and transformed into *E. coli* or other bacterial strains. Effector toxicity can then be determined by inducing the recombinant cloned vector. Following this, coproduction of a given E–I pair will be performed to test whether the putative immunity factor protects against the adverse effects of the toxin [48,52,58,65]. Self-intoxication assays may also be used to verify candidate E–I pairs. The effectors are toxic to bacteria, while their corresponding cognate immunity proteins provide self-resistance. Therefore, a candidate immunity gene mutant is used to test whether its products are able to protect the strain from T6SS mediated antagonism by the wild-type effector in the competition assay, as complementation of the immunity gene mutant eliminates the toxicity of the wild type [34,46,66].

Interaction between E–I pairs may also be verified by means of protein–protein interaction (PPI) methods. For example, through use of a bacterial two-hybrid assay, Tli1^EAEC^ was found directly bound to Tle1^EAEC^ in *E. coli* EAEC 17-2 [51]. Co-immunoprecipitation (Co-IP) studies have been used to study the physical interaction of Tse2 and Tsi2 in *P. aeruginosa* [17], as well as direct interaction between Tli5^PA^ and Tle5^PA^ [16].

Crystal structure analysis can not only verify the directed interaction of E–I pairs, but also provide more detailed information regarding E–I pair complexes. Thus far, a number of E–I pair structures have been revealed, including those of the Tse1-Tsi1 and Tse3-Tsi3 complexes in *P. aeruginosa* [39,43], Tae4-Tai4 complexes in *E. cloacae* and *S. typhimurium* [33], Tae3-Tai3_4_-Tae3 heterohexamers in *R. pickettii* [42], Tge-Tgi complexes in *P. protegens* [13], and Ssp1-Rap1a complexes in *S. marcescens* [47]. 

## 5. Conclusions

T6SS architectures and translocation mechanisms have been elucidated, and the identification of effectors that target both eukaryotic and prokaryotic cells has led to outstanding advances in exploiting of T6SS functions. As the majority of identified effectors exhibit antibacterial activity in a variety of environments [36], E–I pairs appear to be commonly employed in attack and self-defense processes throughout the microbial world. In particular, T6SS-associated E–I pairs play key roles in virulence, either by directly targeting eukaryotic cells by such trans-kingdom effectors, or by indirectly affecting the gut microbiota diversity. In the host environment, E–I pairs might be used by invading pathogens to outcompete other host-associated bacteria, or by commensals to block invading pathogens [36]. Since the immunity proteins could neutralize the toxicity of the toxin effectors, the inactivation of bacterial immunities would lead to killing of pathogens by their own toxins, which would provide novel strategies to combat bacterial infections. Moreover, the antibacterial function of T6SS effectors could be used as an alternative or complementary approach to the use of antibiotics [81].

However, it is likely that only a small part of the T6SS arsenal has been discovered thus far, and many questions remain to be answered. One such question is how the secretion of specific E–I pairs is activated and regulated [6,82]. Although several environmental cues [22,83], transcriptional regulators [68,84], and posttranscriptional regulators [85] have been reported, the T6SS-associated regulatory network remains poorly understood. Another question is these T6SS toxins could kill Gram-negative bacteria, yet no paper has reported the effect of T6SS effectors on Gram-positive bacteria. All these issues need to be studied and clarified in the future. We expect that new E–I pairs from this versatile secretion system will be identified and that new techniques, including software tools, will be employed in their identification. Since offensive and defensive T6SS-associated E–I pairs play a key role in competition for niche space, especially in interbacterial antagonism, and as new E–I pairs have been identified in recent years with some regularity, the prospects for future research into T6SS E–I pairs look promising.

## Figures and Tables

**Table 1:** Identified effector–immunity pairs.

E–I Pairs	Organisms	Effector Activity	Paper Highlights	Citation
**Cell wall Targeting**				
Tse1, 3/Tsi1, 3	*P. aeruginosa*	Amidase (Tse1), Muramidase(Tse3)	Tse1, 3 hydrolyze PG and Tsi1, 3 are the immunity proteins	[32]
Tse1(Tae1)/Tsi1(Tai1)	*P. aeruginosa*	Amidase	Analyzed the crystal structures of Tse1 and Tse1/Tsi1 complex	[39]
Tse3(Tge1)/Tsi3(Tgi1)	*P. aeruginosa*	Hydrolyse PG	Revealed a calcium-dependent membrane-binding mechanism	[43]
Tae1–4/Tai1–4	*B. thailandensis*	Amidase	Defined the Tae superfamily	[14]
Tae3/Tai3	*R. pickettii*	Amidase	Analyzed the structures of Tae3, Tai3 and Tae3/Tai3 complex	[42]
Tae4/Tai4	*E. cloacae*, *S. Typhimurium*	DL-endopeptidase	Analyzed the structure of Tae4/Tai4	[33]
			Proved the cross-immunity of T6SS E–I pairs	[44]
Tae4/Tai4	*S.* *Typhimurium*	Muramidase	Tae4 contributes to bacteria competition and infection	[45]
Tae/Tai	*A. tumefaciens*	Target the PG	Indentified Tae/Tai pairs	[58]
VgrG3/TsaB(TsiV3)	*V. cholerae*	Degrade PG	Identified the VgrG-3 and the antitoxin TsaB	[18]
			Analyzed the structures of native TsaB and the VgrG3C-TsaB complex	[37]
VgrG3/TsiV3(TsaB)	*V. cholerae*	Disrupt bacterial cell wall	Identified E–I pairs with Tn-seq	[12]
Tge1–3/Tgi1–3	*P. protegens*	PG glycoside hydrolase	Identified Tge/Tgi Families	[13]
Ssp1, 2/Rap1a, 2a	*S. marcescens*	Target cell wall	Identified new toxic T6SS pairs	[46]
		PG DL-endopeptidase	Analyzed the E–I pair structures	[47]
Ssp1, 2/Rap1, 2	*S. marcescens*	Predicted amidases	Identified the Ssp1-6 toxins with proteomic method	[48]
TseH/TsiH	*V. cholerae*	Predicted amidase	Identified a new E–I pair with secretome analysis	[72]
**Membrane Targeting**				
VasX/TsiV2, TseL/TsiV1(Tle2/Tli2)	*V. cholerae*	Lipase activity	Identified E–I pairs with Tn-seq	[12]
			The two immunity proteins possess a dual regulatory profile	[50]
Tle1–4, 5(PldA)/Tli1–5	*B. thailandensis*, et al.	Esterases	Discovered a superfamily of bacterial phospholipase	[16]
PldB/PA5088, PA5087, and PA5086	*P. aeruginosa*	Phospholipase D	PldB targets the bacterial periplasm and activate eukaryotic PI3K/Akt pathway	[52]
TplE/TplEi(Tle4/Tli4)	*P. aeruginosa*	Phospholipase A1 and Lipase activity	Toxicity in bacterial periplasm and could induce host cell ER stress and autophagy	[53]
Tle1/Tli1	*E. coli* EAEC 17-2	Phospholipase A1 and A2 activities	The transport of antibacterial Tle1 is mediated by the C-terminus of VgrG	[51]
Hcp-ET2/ETi2 (Tle1/Tli1)	*E. coli* STEC004, *E. coli* PE321	Tle1 Phospholipase	Defined Hcp-ET1-5 and the immunity proteins	[59]
**Nucleotides Targeting**				
Tde1, 2/Tdi1, 2	*A. tumefaciens*	Nucleases	Indentified Tde/Tdi superfamily	[58]
RhsA, B/RhsIA, B	*D. dadantii*	Nucleases	Rhs proteins mediate intercellular competition	[15]
Rhs2-CT/RhsI2	*S. marcescens*	HNH endonuclease	Analyzed the Rhs effectors in intraspecies competition	[34]
Rhs-CT3-8/Rhs-CTI3-8	*E. coli* STEC004, *E. coli* PE027	DNase and RNase	Analyzed the Rhs-CTs family	[20]
Hcp-ET1, 3, 4/ETi1, 3, 4	*E. coli* STEC004, *E. coli* PE321	HNH-DNase (1), Pyocin S3 (3), Colicin-DNase (4)	Defined Hcp-ET1-5 and the immunity proteins	[59]
Tke2/Tki2	*P.* *putida*	Nucleases	Toxic Rhs-type effectors were identified and characterized	[57]
**Other effectors**				
Hcp-ET5/ETi5	*E. coli* STEC004, *E. coli* PE321	Papain-like peptidase	Defined Hcp-ET1-5 and the immunity proteins	[59]
Tse2/Tsi2	*P. aeruginosa*	Arrest bacteria growth	Identified Tse1–3 effectors and immunity protein Tsi2	[17]
Tse2/Tsi2	*P. aeruginosa*	Induce bacterial quiescence	Structure analysis revealed the interaction mechanism of Tse2/Tsi2	[60]
Tse2/Tsi2	*P. aeruginosa*	NAD-dependent ADP-ribosylating toxins	Analyzed the structure of Tse2 and Tsi2	[61]
Tse4-6/Tsi4-6	*P. aeruginosa*	Antibacterial effectors	Proteomics screen for T6SS substrates	[62]
Tse6/Tsi6	*P. aeruginosa*	NAD(P)+ Glycohydrolase	Analyzed the function, delivery and structure of Tse6 toxin	[64]
Tke1, 3/Tki1, 3	*P.* *putida*	NAD(P)+ Glycohydrolase (Tke1), unknown for Tke3	Toxic Rhs-type effectors were identified and characterized	[57]
TseC/TsiC	*A. hydrophila*	Antibacterial toxicity with a predicted colicin domain	Identified T6SS effector using a conserved chaperone domain	[67]
RhsP1, 2-CT/RhsI1, 2	*P. aeruginosa*	Antibacterial toxicity	Identified new E–I pairs carried by VgrG	[65]
Rhs-CT1, 2, 9/Rhs-CTI1, 2, 9	*E. coli* STEC004, *E. coli* PE027	Metallopeptidase (1, 2) or Deaminase (9)	Analyzed the Rhs-CTs family	[20]
Bfe1, 2/Bfi1, 2	*B. fragilis*	Antagonism function	T6SS E–I pairs were responsible for antagonism to gut Bacteroidales species	[66]
